# Sneaky emotions: impact of data partitions in affective computing experiments with brain-computer interfacing

**DOI:** 10.1007/s13534-023-00316-5

**Published:** 2023-09-11

**Authors:** Yoelvis Moreno-Alcayde, V. Javier Traver, Luis A. Leiva

**Affiliations:** 1 Institute of New Imaging Technologies, Universitat Jaume I, Av. Vicent Sos Baynat, s/n, Castellón, 12071 Castellón Spain; 2 University of Luxembourg, Esch-sur-Alzette, Luxembourg

**Keywords:** Emotion recognition, Videos, BCI, EEG, Data splits

## Abstract

Brain-Computer Interfacing (BCI) has shown promise in Machine Learning (ML) for emotion recognition. Unfortunately, how data are partitioned in training/test splits is often overlooked, which makes it difficult to attribute research findings to actual modeling improvements or to partitioning issues. We introduce the “data transfer rate” construct (i.e., how much data of the test samples are seen during training) and use it to examine data partitioning effects under several conditions. As a use case, we consider emotion recognition in videos using electroencephalogram (EEG) signals. Three data splits are considered, each representing a relevant BCI task: subject-independent (affective decoding), video-independent (affective annotation), and time-based (feature extraction). Model performance may change significantly (ranging e.g. from 50% to 90%) depending on how data is partitioned, in classification accuracy. This was evidenced in all experimental conditions tested. Our results show that (1) for affective decoding, it is hard to achieve performance above the baseline case (random classification) unless some data of the test subjects are considered in the training partition; (2) for affective annotation, having data from the same subject in training and test partitions, even though they correspond to different videos, also increases performance; and (3) later signal segments are generally more discriminative, but it is the number of segments (data points) what matters the most. Our findings not only have implications in how brain data are managed, but also in how experimental conditions and results are reported.

## Introduction

Recently, a lot of research effort is being paid to Affective Computing [29, 36] in general, and to Brain-Computer Interfaces (BCI) [3, 40] in particular, within the context of emotion recognition with Machine Learning (ML) models. Specifically, researchers have proposed many approaches to collect, analyze, and model electroencephalogram (EEG) signals, with promising results in terms of classification performance; e.g. [1, 4, 5, 24, 25, 30, 39]. Furthermore, with the advent of Deep Learning, more advanced ML models have been proposed over the last few years, with sometimes impressively high recognition performance results being reported. However, unlike what happens in, for example, the Computer Vision community (e.g. [9, 23, 26, 28]), there is a lack of shared protocols and benchmarking practices in the BCI community, which makes the proposed approaches hardly comparable and does not promote or ensures the correctness of a given model or technique. Furthermore, quite often the described experimental methodology lacks details or is ambiguous, which leaves us wonder to what extent the reported performance results have been achieved under fair experimental conditions. Eventually, this status quo does not help researchers with building up on previous work nor selecting the most adequate modelling technique. Therefore, raising awareness of these issues can contribute to improved research practices as well as clearer and more realistic expectations of the potential and current limitations of BCI-based emotion recognition.

Certainly, emotion recognition using BCI signals is a challenging problem, especially when it comes to understanding affective responses towards dynamic contents such as videos, mainly because of the high inter-subject and intra-subject variability [37] and the dynamic nature of videos [18]. Part of these difficulties are lately addressed with techniques such as contrastive learning [34] or domain adaptation [8], following the common idea of explicitly bringing together learned representations of brain signals corresponding to similar emotional responses, even though coming from different subjects.

In the literature, three data regimes are typically considered in affective modeling problems: subject-dependent, subject-independent, and cross-subject. *Subject-dependent* is considered the most favorable condition, since a personalized ML model is trained on subject-specific data and only data from the very same subject is used for testing the model; so usually the highest performance is achieved under this condition. In the subject-independent case, however, a single model is learned with data from several subjects, who are combined during training and testing. *Subject-independent* is considered more challenging but also more realistic than the subject-dependent regime. Correspondingly, the reported model performance is usually lower. However, how much data from one subject is used in training is critical to understand whether the merits of the achieved performance corresponds to the generalization ability of the proposed ML model or to the amount of the data from test subjects that has been seen during model training. Finally, the *cross-subject* scenario is considered the hardest and most useful in practice, since the ML models are tested on data from subjects that were never seen in model training.

Another critical factor that makes emotion recognition using BCI signals a challenging problem is the size of the datasets. BCI datasets are usually small in size, due to the cost of acquiring these signals. This has an impact on the kind of ML models that can be used, since, for example, (deep) neural networks typically require lots of training instances to avoid overfitting. To alleviate this issue, researchers have considered different temporal segments (or chunks) of the BCI signals as independent data points for ML model development. While this certainly helps to increase the number of training and testing samples, there is a potential data leakage issue because neighboring segments are expected to be similar. Therefore, ML models are tested on samples that are very similar to those seen during training. This problem is further exacerbated when those segments overlap.

In this paper, we provide a rigorous analysis of these data partitioning issues. We introduce the “data transfer rate” construct (i.e., how much data of the test samples are seen during model training) and use it to examine data partitioning effects under several conditions. As a use case, we consider EEG signals and videos as input stimuli. First, we study subject-independent data splits, which is relevant for generalized ML models of affective decoding. Second, we study video-independent data splits, which is relevant for affective annotation of multimedia contents. Third, we study time-based data splits, which is relevant for preprocessing and feature extraction in ML. Taken together, our results show that (1) for affective decoding, it is hard to achieve recognition performance above the baseline case (random classification) unless some data of the test subjects are considered in the training partition; (2) for affective annotation, having data from the same subject in training and test partitions, even though they correspond to different videos, slightly increases performance; and (3) later signal segments are generally more discriminative, but it is the number of segments (data points) what matters the most to improve performance. Our findings not only have implications in how BCI signals are managed, but also in how experimental conditions and results are reported in academic papers.

### Related work

The following literature overview is not meant to be exhaustive, given the large body of research existing on emotion recognition with BCI devices, but to illustrate the different reported model performances in order to contextualize the results yielded later in our analysis. As indicated before, we consider EEG signals and videos as input stimuli. We focus on a very popular dataset (DEAP) [19] and on the most popular ML task: binary classification of valence [22, 32, 33]. Valence is a positive or negative quantification of affective appraisal, or the degree an emotion has a pleasant or unpleasant quality [12].

In subject-independent experiments, $89.83\%$ accuracy is reported by Galvão et al. [13] using a *k*-NN regressor in a 10-fold cross-validation setting. Keelawat et al. [17] tested Convolutional Neural Networks (CNNs) of 3–7 layers and achieved $86.87\%$ accuracy with 6 layers and 10-fold cross validation, and $68.75\%$ accuracy with 4 layers and leaving-one-subject out. Yin et al. [39] combined graph-based CNNs and long short-term memory (LSTM) cells, achieving $84.81\%$ accuracy. Huang et al. [16] developed a CNN that exploited the differences in patterns between the left and right brain hemispheres, achieving $68.14\%$ accuracy. Du et al. [8] applied attention to the output of LSTM for the automatic selection of the emotion-relevant EEG channels, and obtained $69.06\%$ acccuracy. Classification accuracy higher than $99\%$ is reported with a combination of a Deep CNN (DCNN) and a Support Vector Machines [30]. With a spatio-temporal-spectral network, an accuracy of $69.38\%$ is obtained [21]. Finally, Xu et al. [38] reported an accuracy of $67.36\%$ using a combination of Gated Recurrent Unit (GRU) cells and a CNN.

Towards the ideal scenario of callibration-free emotion recognition, where no brain data from a target subject would be required in advance, a few-shot learning study by Bhosale et al. [4] reported average few-shot classification accuracy ranging from $67.24\%$ (under 5-shot and random sampling) to $78.12\%$ (under 25-shot and subject-dependent sampling). In a zero-calibration setup, accuracy ranged from $62.98\%$ (5-shot, subject-dependent) to $71.68\%$ (25-shot and subject-independent).

In cross-subject experiments, an average accuracy of $79.99\%$ has been reported by Gupta et al. [14]. Liu et al. [24] explored domain adaptation through subject clustering, achieving an accuracy of $73.9\%~(\pm 13.54\%)$.

**Table 1:** Binary valence classification performance on DEAP dataset over the last 5 years

Year	Accuracy (%)	Subject-independent	Cross-subject	ML model
2019	79.99		[14]	Random forest, SVM
2019	86.87	[17]		CNNs
2019	68.75	[17]		CNNs
2021	84.81	[39]		Graph-based CNN + LSTM
2021	73.9		[24]	Clustering + neural network
2021	89.83	[13]		*k*-NN regressor
2021	68.14	[16]		CNN
2022	67.24	[4]		3D CNN + LSTM
2022	78.12	[4]		3D CNN + LSTM
2022	69.06	[8]		LSTM
2023	99.31	[30]		Deep CNN + SVM
2023	69.38	[21]		ManifoldNet + LSTM
2023	67.36	[38]		GRU + CNN

While these results provide a rough idea of the performance range in state-of-the-art methods, it also highlights a significant variability between them and an unclear trend along the years (Table 1). This means that it is difficult to understand the relationship between model complexity and achieved performance. It is therefore hard to judge whether the performance differences are attributed to either improvements in data preprocessing or feature extraction techniques, or to the particular ML approach, or to the data splits used. To shed more light in this regard, in this work we consider constant the data processing and the ML model, and conduct a careful analysis on the relationship between the data splits and recognition performance.

## Materials and methods

### Dataset and setup

We conducted our experiments on the DEAP dataset [19], which is perhaps the most popular dataset for the analysis of human affective states. Relevant to our research, DEAP provides EEG signals (32 channels) of 32 participants while watching 40 one-minute long excerpts of music videos. Participants rated each video in terms of valence, arousal, like/dislike, dominance, and familiarity. DEAP includes both raw and preprocessed EEG signals. In our experiments, we use the latter to ease replication and comparisons against previous work. Preprocessing includes downsampling the 512 Hz original signal to 128 Hz, removing electrooculography artefacts, and applying a band-pass filter in the [4,45] Hz range.^1^

We divided the one-minute brain signals into short temporally consecutive segments of 1, 2, or 4 s long, without overlap.^2^ DEAP includes a 3-second long EEG signal previous to the stimulus. Following [16], the average of the three one-second segments of this “rest-state” signal results in 32-D mean vector per channel, which is subtracted, also channel-wise, to each 1-second segment of the EEG signal during the presentation of the visual stimulus. Then, each channel is separately scaled to have unitary maximum absolute value.

Each pair (*v*, *s*) of video $v\in \{1,\ldots ,40\}$ and subject $s\in \{1,\ldots ,32\}$ has a label $\ell \in [1,9]$ for each emotional dimension (valence, arousal, and dominance), which corresponds to the subjective self-reported annotation. In this work, we considered only the valence dimension, and binarized its values into “negative” ($\ell \le 5$) and “positive” ($\ell > 5$), in line with much of previous work  [22, 32, 33]. Therefore, we consider a 2-way (binary) classification problem. The binarized labels are used as ground-truth for model training and performance evaluation on the test samples. Each individual segment inherits the label from the (*v*, *s*) signal it belongs to.

### Data splits

We consider splits data at three different target levels: subject-level, video-level, and time-level. In the first two cases, we consider a data transfer rate that represents how much data of the test target, expressed as the ratio $\beta \in [0,1]$, is “transferred” to the training partition. Regarding affective content analysis, two tasks commonly considered are affective *decoding* and affective *annotation* [31]. Affective decoding refers to the estimation of the emotional response to a given stimulus for a particular subject, whereas affective annotation involves attaching descriptive affective metadata to digital contents (e.g. assigning an automatically predicted emotional response to a given image) for subsequent use (e.g. in affective-based content retrieval). Accordingly, three types of data splits of the segmented brain signals were considered, each corresponding to a different practical scenario:

- *Subject-independent splits* are relevant to affective decoding settings. Data from a random subset of subjects were used for training and a disjoint subset of subjects was used for testing.
- *Video-independent splits* are relevant to affective annotation tasks. Data from a random subset of videos were used for training and a disjoint subset of videos was used for testing.
- *Time-based sampling* represents practical scenarios in BCI recording sessions or live interaction contexts. Here, the last 20% duration of each video (i.e., 60/5 = 12 s) was used for testing, since there is some evidence that the last part of the brain signal is more relevant [20]. The remaining 80% was used as a pool of samples to be added to the training partition, as detailed below.

For subject-independent and video-independent cases, the respective procedures described above were repeated following *k*-fold cross-validation. We used $k=5$ since it represents a good choice for moderate computational complexity and test size representativeness. Note that a larger *k* would imply smaller test sets and more training rounds.

**Fig. 1:**
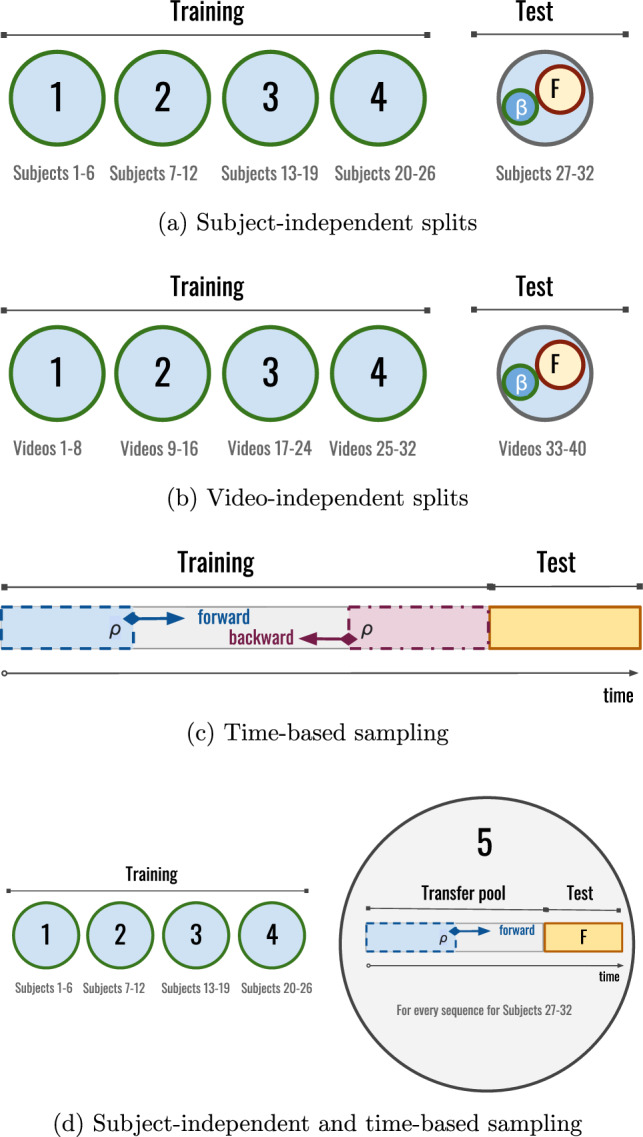
Schematics of the different data splits considered in this work. In **a**, **b**, and **d** the numbers of subjects and videos within each fold are sorted for presentation simplicity, but random disjoint subsets were actually considered in our experiments

The data splits were determined as follows (Fig. 1). The size of the test set was fixed to $F$, a ratio of the total dataset size. Importantly, this size remains the same regardless of the transfer rate $\beta$, which guarantees that the test set is not a confounding variable and, therefore, the effects on the dependent variable (performance) are only attributable to the independent variable ($\beta$). Another important detail is that the pool of the test set used for data transfer rate is disjoint to the test set actually used for performance evaluation, so $\beta$ applies only to the remaining *R* (%) test samples. The transfer rate was varied as $\beta \in \{0,0.2,0.4,0.6,0.8\}$.

Take for example the 5-fold example shown in Fig. 1a and b. Since $k=5$, if $F=4$%, for each of the $k=5$ folds, the test fold has $100/k=20\%$ of the data samples, from which a global amount $F$ is fixed for testing, and different amounts of the remaining $R=20-F$ (%) are used to take different $\beta$ ratios. Thus, if $F=4\%$, then $R=20-4=16\%$. Therefore, with $\beta =0.2$, a total of $\beta \cdot R = 0.2\cdot 16\%=3.2\%$ of the total samples are additionally included in the training set. For the subject-independent experiments (Fig. 1a), since there are $S=32$ different subjects in the dataset, each fold has data of *S*/*k* different subjects (i.e. 6 or 7 subjects per fold). Notice that the particular subset of test instances per each fold is fixed, so that it is not affected by $\beta$.

For the time-based sampling experiments, we analyze the influence of the temporal provenance of signal segments. We increasingly chose different segment lengths following either a forward or backward strategy. Concretely, for each EEG sample ${{\textbf {x}}}_{1:T}$ in the training set, segments from increasingly longer parts of the subsequence ${{\textbf {x}}}_{1:\rho T}$ are considered for training in the forward case, and ${{\textbf {x}}}_{T(1-\rho ):T}$ for the backward case, as illustrated in Fig. 1c. The sequence ratio $\rho$ was varied as $\rho \in \{0.2, 0.4, 0.6, 0.8\}$.

### Machine learning model

We used our own PyTorch Lightning [10] implementation of a CNN model based on MIN2Net [2], which is an architecture proposed for motor-imagery tasks. MIN2Net implements a multi-task learning framework with three additive losses: supervised classification, reconstruction, and metric learning components. Our architectural choice relies on the fact that, compared to other proposed models, MIN2Net provides an excellent balance between complexity and performance, which seems more suitable given the limited amount of available data, as usual in most of today’s BCI datasets. We explored several variants of MIN2Net associated to different combinations of the above-mentioned three loss functions, but did not observe notable differences; thus we report our results using the classification loss only.

The input to our model is a tensor of size $C \times L$ where *C* is the number of BCI channels (32 in DEAP), and $L=f\cdot T$, with *T* being the duration of a signal segment and *f* the sampling frequency (128 Hz in DEAP). The architecture is depicted in Table 2.

**Table 2:** The CNN network we used consists of two convolutional blocks and a classification block

Layer	
Conv2D(n:512, k:$1\times 65$ , s:$1\times 1$ )	
ELU($\alpha$ :1.0)	
BatchNorm2D($\epsilon$ :$10^{-5}$ , *m* :0.1)	
AvgPool2D(k:$1\times 2$ , s:$1\times 2$ )	
.	
Conv2D(n:10, k:$1\times 33$, s:$1\times 1$ )	
ELU($\alpha$ :1.0)	
BatchNorm2D($\epsilon$ :$10^{-5}$ , *m* :0.1)	
AvgPool2D(k:$1\times 4$ , s:$1\times 4$ )	
.	
Flatten	
Linear(n:160)	
BatchNorm1D($\epsilon$ :$10^{-5}$ , *m* :0.1)	
ReLU	
Linear(n:1)	

For convolutional and pooling layers, *k* is the kernel size and *s* is the stride. In convolutional layers, *n* is the number of filters. In fully connected (linear) layers, *n* is the number of hidden units. In batch normalization layers, *m* is the momentum

The binary cross-entropy was used as classification loss. The batch size was 100 temporal segments. The model was trained up to 15 epochs, but the model with lowest validation loss was kept for testing. The optimizer was Adam with a linear scheduled learning rate $\gamma =10^{-3}$ (warm up of 10%), weight decay $\lambda =0.01$, and parameters $\beta _1=0.9,\beta _2=0.999$.

## Results

### Subject-independent tests

**Fig. 2:**
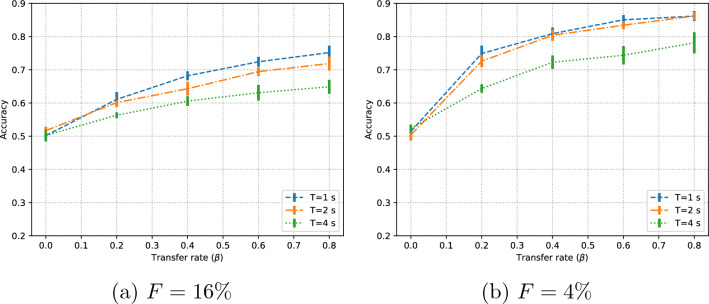
Effect of data transfer rate in subject-independent tests with two different sizes $F$

The influence of data transfer rate (Fig. 2) is clear: with no data transfer rate ($\beta =0$) the model performance is essentially random. Then, with increasing $\beta$, classification performance increases steadily. The effect is stronger with shorter signal segments, despite the fact that short segments carry less information and thus could be considered less discriminative. The likely reason for this behavior is two-fold: shorter segments imply more training instances, and these instances are more likely to be similar in the training and test sets. For an alternative, leaving-one-subject-out validation, results are similar (Appendix A).

The effect of different amounts of $F$ for the fixed test set can be seen by comparing Fig. 2a and b. Complementary confusion matrices are given in Table 3. These subfigures represent two testing conditions: on the one hand, a large $F$ is important to have a representative test set; on the other hand, smaller $F$ implies more training data to choose from (i.e. $\beta \cdot R$ is higher). Then, classification performance with $F=4\%$ is significantly higher. Similar trends were observed in a traditional classifier (Appendix. B). This highlights the importance of properly conducting and reporting the data splits in academic papers. Without a shared protocol and further information, the performance reported in Fig. 2b suggests that the method is preferable over that of Fig. 2a even though they correspond to exactly the same method and ML model; only the test set and data transfer rate are different.

**Table 3:**
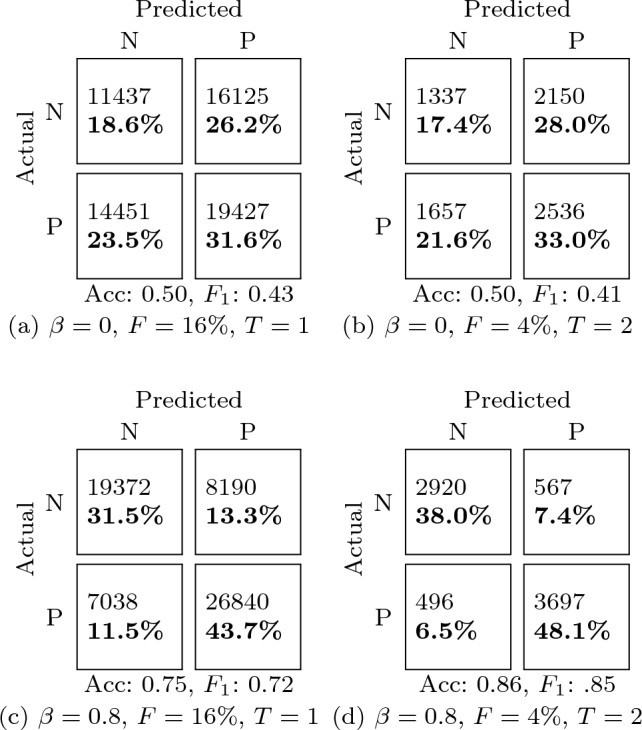
Confusion matrices for the minimum (a, b) and maximum (c, d) mean accuracy for the subject-independent tests (Fig. 2)

N and P  stand for the “negative” and “positive” valence classes. Each cell includes the number of test instances and the corresponding overall percent. Below the matrix, the accuracy (Acc) and $F_1$ score are included

It is important to highlight that the performance improvement is mainly due to the data from subjects in the test set being used in training, not simply because more training data is being used. As an evidence of this fact, the performance achieved at $\beta =0$ with 5-fold and 10-fold (not shown here) is essentially the same (random performance) in spite of having twice as many training data samples in the 5-fold case (20% of the dataset) than in the 10-fold case (10% of the dataset).

**Fig. 3:**
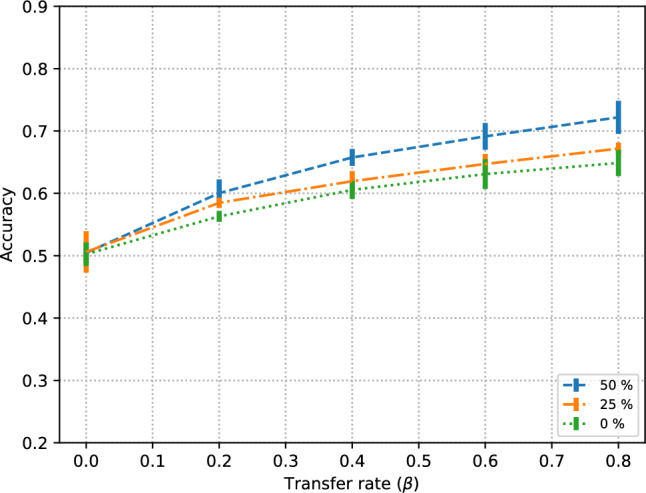
Effect of data transfer rate in subject-independent experiments $F=16\%$ with three segment overlap ratios: no overalap (0%), 25% and 50%

We should note that the temporal segments used in these experiments do not overlap. Results for signal segments of $T=4$ s with 25% overlap (i.e. 1 s) and 50% overlap (i.e. 2 s) illustrate the notable performance improvement (Fig. 3), with classification performance comparable to those of $T=2$ s or $T=1$ s without overlap. It is important to note that overlapping segments can be seen as one of the strongest forms of data leakage. Therefore it is generally advisable not to use them if we care about model generalization.

### Video-independent tests

**Fig. 4:**
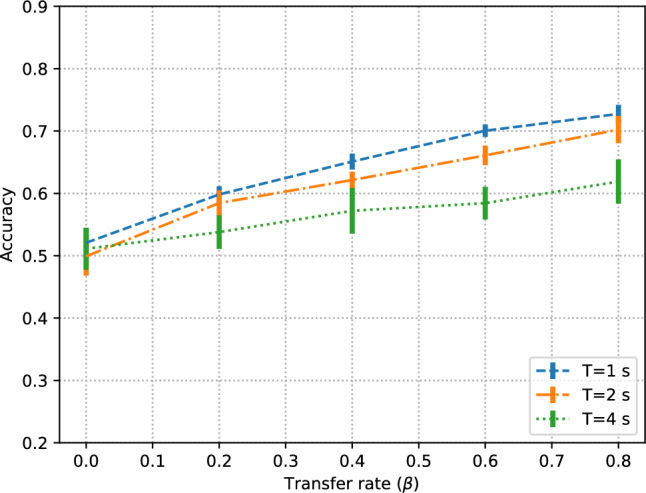
Effect of data transfer rate in video-independent experiments

For the video-independent case, similar trends (Fig. 4) to those observed in the subject-independent tests, happen in terms of data transfer rate ($\beta$). See confusion matrices (Table 4) for complementary information. In absolute terms, the average performance for a given $\beta$ is slightly higher in the video-independent cases than in the subject-independent cases. A sensible explanation is that even though EEG data from a test video is not seen in the training set, there are data from the same subject in the training and test sets, albeit corresponding to different videos. Therefore, although EEG data is both subject-specific and video-specific, the information specific to one subject is slightly harder to generalize and, therefore, classification performance in the subject-independent tests is a bit lower.

**Table 4:**
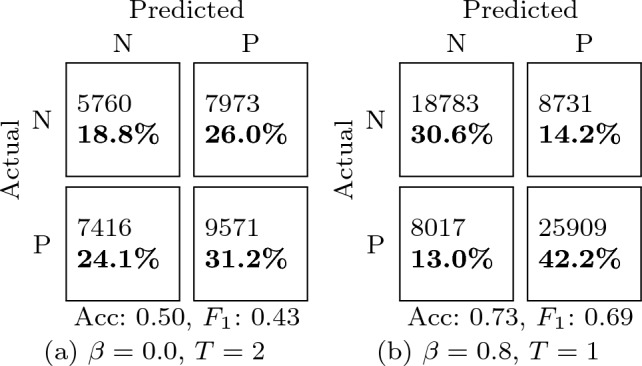
Confusion matrices for the video-independent tests (Fig. 4) corresponding to the minimum (left) and maximum (right) mean accuracy

### Time-based sampling

**Fig. 5:**
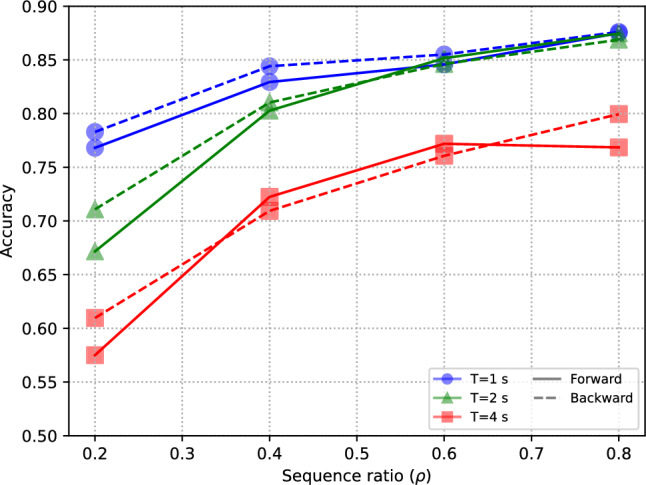
Effect of forward and backward temporal sampling for three different segment lengths *T*

In the time-based splits, classification performance is notably higher overall (Fig. 5, Table 5) than in the subject-independent and video-independent tests, since in this case the segments corresponding to the same subjects and videos are both in the training and test splits, since the focus of these experiments was on the timestamp of the segments. It is apparent that the length of the segment has an impact even higher than in subject-independent or video-independent tests, with higher performance being obtained with shorter segments. This can be explained by the fact that short segments that are temporally contiguous are more likely to be similar than longer segments.

Finally, the performance differences between forward and backward temporal sampling is only noticeable at the smallest training sizes considered ($\rho =0.2$). This suggests that having training data corresponding to the last part of the brain signal has a higher discriminative power at small-data regimes, but this effect tends to be less relevant than the amount of training data.

**Table 5:**
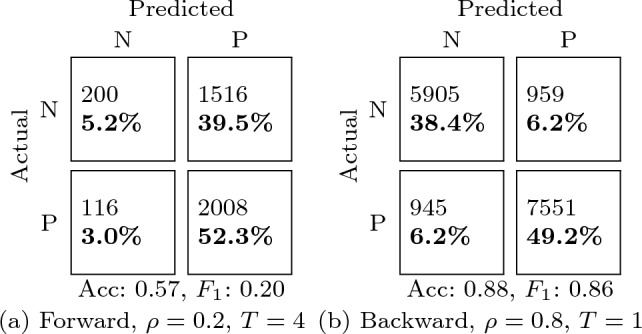
Confusion matrices for the temporal sampling tests (Fig. 5) corresponding to the minimum (left) and maximum (right) accuracies

### Subject-independent and time-based sampling

**Fig. 6:**
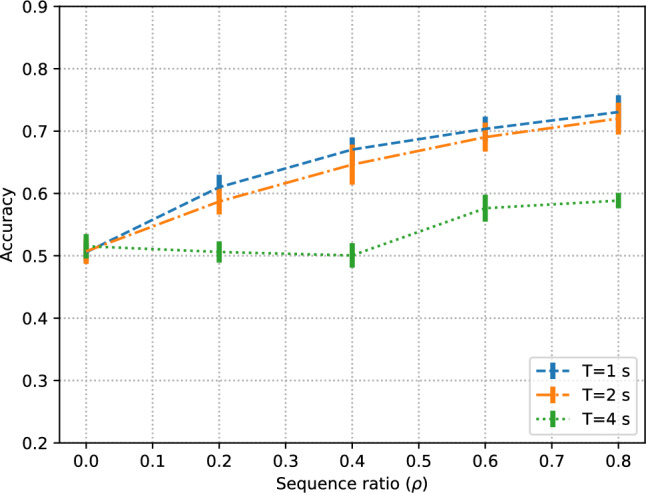
Combined effect of forward temporal sampling and data transfer rate in subject-independent experiments for $F=16\%$

Finally, in light of the previous results, we combine the data transfer rate within the subject-independent scenario with (forward) temporal sampling. Figure 6 indicates that classification performance is slightly worse than those observed in the subject-independent experiments (Fig. 2) because the imposed temporal constraint of the temporal segments (increasing $\rho$ correspond to more increasingly later segments being used) makes the segments in the training and test segments less similar at lower $\rho$. With respect to (subject-agnostic) temporal sampling (Fig. 5), classification performance is remarkably smaller because the amount of data from the same subject is more limited. The result is particularly lower for $T=4$ s since there are fewer training segments of that length and many more (i.e. higher $\rho$) are required to better help discriminating emotions.

## Discussion

Over the last few years, very good classification performance has been reported in BCI-based emotion recognition experiments, especially when using EEG signals. However, previous work is often unclear about the experimental protocol and, importantly, the data splits used. We have looked into this problem and empirically studied the impact on performance of experimental details regarding data partitioning. Although our experiments have been performed on a single dataset and one neural architecture, our findings suggest how critically important the details about data splits are. Specifically, variations in accuracy ranged from about random ($\approx 50\%$) to nearly $90\%$ using the very same ML model. This calls for more attention when conducting BCI experiments and reporting results, especially under subject-independent and cross-subject protocols. We strongly believe that a shared definition of these different protocols should also be explicitly acknowledged in the published works. Authors should be diligent in providing those important details unambiguously, which should be promoted or ensured by our peers.

We have worked on valence classification based on its popularity among the BCI community. Since valence and arousal can have different temporal dynamics [6], the temporal-based sampling test was repeated for the arousal dimension. We found similar recognition trends as in valence, although the performance benefit of backward over forward temporal sampling holds for larger sequence ratios (up to $\rho =0.4$) for the longest segment ($T=4$), which might suggest that the identification of the arousal level is more dependent than the valence on the late temporal portion of the brain signal.

Our work can be extended to more than two classes, other emotion dimensions (e.g. arousal and dominance), and other learning tasks (e.g. regression instead of classification). It is also assumed, as done in the research literature, that separately classifying temporal segments of the entire brain signal is a meaningful approach. However, while being exposed to dynamic contents such as videos, the assumption that all segments carry the same sequence-level affective information might need to be revisited [22]. Another direction to look into is to what extent data augmentation techniques may alleviate the lack of target-specific (video, subject) data.

Future work should revisit how ground-truth information is constructed. Typically, participants in BCI studies report self-perceived measures of affective states (e.g. valence or arousal values in a graded scale). Given the variability of the BCI signals in response to dynamic stimuli, it can also be argued how much of the participant’s self-reported response is actually present in each of these (short) segments which the BCI signals are typically split into.

Our findings can be summarized in terms of three key scenarios explored: subject-independence, video-independence, and temporal sampling, which in turn relate to three important BCI research topics, namely, affective decoding, affective annotation, and brain signal recording sessions and usage.

Affective decoding With no subject-specific information included in the training set, classification performance is expected to be essentially random, at least in the small data regime (which is the case in the majority of BCI studies). Then, performance should quickly increase with an increasing data transfer rate. This means that even for powerful state-of-the-art ML models, it is hard to learn features that generalize to unseen subjects. The practical implications is that calibration-free BCI is essentially not possible as of today. Interestingly, with a few data samples from the target subject, performance increases. This suggests that a short calibration stage might be helpful, in order to collect such little but valuable data.

Affective annotation For stimulus-level analysis, the results are similar to the subject-independent case. In practice, this means that annotating new contents, for which no emotional response has yet been observed during training, is a really challenging endeavor. As soon as some signal segments from a target video are available during training, classification performance increases progressively. This is more remarkable with shorter segments.

Temporal sampling When temporal segments are used in the training set, according to their timestamp, we found that using segments later in the sequence provides diminishing returns in terms of model recognition. The practical implication of this finding is that shorter capture sessions might be enough and that a favourable tradeoff between recognition performance and human effort is possible. For example, in the one-minute video stimuli of the DEAP dataset, about 40% of the length of the signals sequences (corresponding to about 20 s) may already provide high-rate affective decoding using one-second length segments if multiple subjects are considered. Although this requires a set of participants, it reduces the effort per participant to provide brain data. On the other hand, our results suggest that short segments carry sufficiently discriminative information, which implies that on-line learning algorithms might be used at training or deployment time, without incurring in a significant delay to wait for subsequent parts of the brain signals to be captured and processed.

### Conclusion

We have investigated the effect of data splits in binary valence classification performance, and found significant differences in several practical scenarios. This effect has been largely overlooked in the research literature; therefore it is difficult to attribute previous research findings to actual modeling improvements or to data partitioning issues. Our findings not only have implications in how BCI signals are managed, but also in how experimental conditions and results are to be reported in academic papers.

## Figures and Tables

**Fig. 7:**
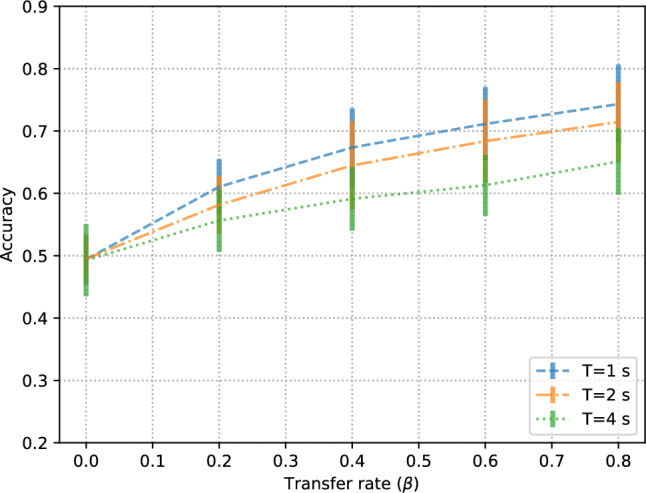
Effect of data transfer rate in subject-independent tests using leaving-one-subject-out

**Fig. 8:**
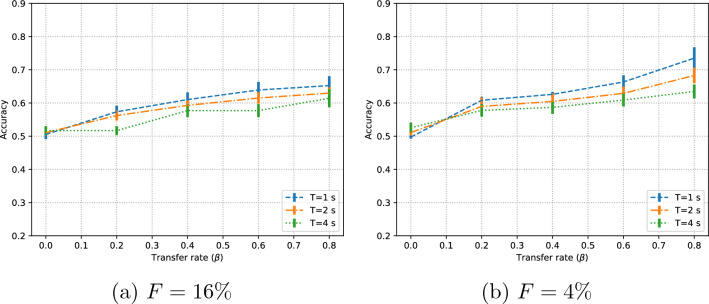
Effect of data transfer rate in subject-independent tests with two different sizes $F$ for a *k*-NN classifier

## A. Leaving one subject out

For subject-independent tests, the leaving-one-subject-out (LOSO) validation can be an alternative to consider, in particular when data is scarce and/or data from a given subject should only be considered in the test set. We tested the effect of the data transfer rate under this LOSO setting, and the result with $F=2.5\%$ (Fig. 7) is similar to the 5-fold analysis using also 80% of the test fold (Fig. 2a) for the fixed part (*F*). The higher variance in the LOO case can be due to having more, and more diverse, test sets.

## B. Traditional classifier

To further understand to what extent the effect on performance of data partitioning is similar in traditional ML classifiers, we used a *k*-nearest neighbor, *k*-NN ($k=4$, distance-based neighboring voting weights, and $L_1$ metric, ties resolved by the order in the training set). As hand-crafted features, the Fast Fourier transform (FFT) was applied over each temporal segment of length *T*, and then five features (three Hjorth parameters [15], spectral entropy [7], and signal energy [11]) were computed per channel and frequency band, resulting in $5~\text {features} \times 32~\text {channels} \times 5~\text {bands} = 800$ features. The settings for the classifier and the features are based on existing literature [27, 35, 41].

Results on subject-independent tests (Fig. 8) indicate that the trends are similar to those of a CNN-based model (Fig. 2), although performance improves at a lower rate with increasing data transfer rates, which can be explained by the significantly different nature of the machine-learning models.

## Footnotes

1 See http://www.eecs.qmul.ac.uk/mmv/datasets/deap/readme.html.

2 Except in one of our experiments where we explore the effect of overlapping segments.
